# Global, Race-Neutral Reference Equations and Pulmonary Function Test Interpretation

**DOI:** 10.1001/jamanetworkopen.2023.16174

**Published:** 2023-06-01

**Authors:** Alexander T. Moffett, Cole Bowerman, Sanja Stanojevic, Nwamaka D. Eneanya, Scott D. Halpern, Gary E. Weissman

**Affiliations:** 1 Division of Pulmonary, Allergy, and Critical Care Medicine, Department of Medicine, University of Pennsylvania, Philadelphia; 2 Palliative and Advanced Illness Research (PAIR) Center, University of Pennsylvania, Philadelphia; 3 Leonard Davis Institute of Health Economics, University of Pennsylvania, Philadelphia; 4 Department of Community Health and Epidemiology, Dalhousie University, Halifax, Nova Scotia, Canada; 5 Michael G. DeGroote School of Medicine, McMaster University, Hamilton, Ontario, Canada; 6 Renal-Electrolyte and Hypertension Division, Department of Medicine, University of Pennsylvania, Philadelphia; 7 Department of Biostatistics, Epidemiology, and Informatics, University of Pennsylvania, Philadelphia; 8 Department of Medical Ethics and Health Policy, University of Pennsylvania, Philadelphia

## Abstract

**Importance:**

Race and ethnicity are routinely used to inform pulmonary function test (PFT) interpretation. However, there is no biological justification for such use, and it may reinforce health disparities.

**Objective:**

To compare the PFT interpretations produced with race-neutral and race-specific equations.

**Design, Setting, and Participants:**

In this cross-sectional study, race-neutral reference equations recently developed by the Global Lung Function Initiative (GLI) were used to interpret PFTs performed at an academic medical center between January 2010 and December 2020. The interpretations produced with these race-neutral reference equations were compared with those produced using the race and ethnicity–specific reference equations produced by GLI in 2012. The analysis was conducted from April to October 2022.

**Main Outcomes and Measures:**

The primary outcomes were differences in the percentage of obstructive, restrictive, mixed, and nonspecific lung function impairments identified using the 2 sets of reference equations. Secondary outcomes were differences in severity of these impairments.

**Results:**

PFTs were interpreted from 2722 Black (686 men [25.4%]; mean [SD] age, 51.8 [13.9] years) and 5709 White (2654 men [46.5%]; mean [SD] age, 56.4 [14.3] years) individuals. Among Black individuals, replacing the race-specific reference equations with the race-neutral reference equations was associated with an increase in the prevalence of restriction from 26.8% (95% CI, 25.2%-28.5%) to 37.5% (95% CI, 35.7%-39.3%) and of a nonspecific pattern of impairment from 3.2% (95% CI, 2.5%- 3.8%) to 6.5% (95% CI, 5.6%-7.4%) and no significant change in the prevalence of obstruction (19.9% [95% CI, 18.4%-21.4%] vs 19.5% [95% CI, 18.0%-21.0%]). Among White individuals, replacing the race-specific reference equations with the race-neutral reference equations was associated with a decrease in the prevalence of restriction from 22.6% (95% CI, 21.5%-23.6%) to 18.0% (95% CI, 17.0%-19.0%), a decrease in the prevalence of a nonspecific pattern of impairment from 8.7% (95% CI, 7.9%-9.4%) to 4.0% (95% CI, 3.5%-4.5%), and no significant change in the percentage with obstruction from 23.9% (95% CI, 22.8%-25.1%) to 25.1% (95% CI, 23.9%- 26.2%). The race-neutral reference equations were associated with an increase in severity in 22.8% (95% CI, 21.2%-24.4%) of Black individuals and a decrease in severity in 19.3% (95% CI, 18.2%-20.3%) of White individuals vs the race-specific reference equations.

**Conclusions and Relevance:**

In this cross-sectional study, the use of race-neutral reference equations to interpret PFTs resulted in a significant increase in the number of Black individuals with respiratory impairments along with a significant increase in the severity of the identified impairments. More work is needed to quantify the effect these reference equations would have on diagnosis, referral, and treatment patterns.

## Key Points

**Question:**

How do race-neutral reference equations affect pulmonary function test interpretation?

**Findings:**

In this cross-sectional study of 8431 Black and White individuals, the use of race-neutral reference equations for pulmonary function test interpretation, compared with the race-specific reference equations that are currently recommended, resulted in an increase in the percentage of Black individuals identified with restrictive ventilatory impairments from 26.8% to 37.5% and an increase in the percentage of Black individuals identified with nonspecific ventilatory impairments from 3.2% to 6.5%. The percentage of White individuals identified with restrictive impairments decreased from 22.6% to 18.0%, while the percentage of White individuals identified with nonspecific impairments decreased from 8.7% to 4.0%.

**Meaning:**

In this study, the use of race-neutral reference equations was associated with an increase in the prevalence and severity of ventilatory impairments in Black patients.

## Teaser

This cross-sectional study evaluates the association of race-neutral reference equations developed by the Global Lung Function Initiative with the interpretation of pulmonary function tests among Black and White individuals.

## Introduction

Pulmonary function test (PFT) interpretation involves the comparison of observed and predicted measures of pulmonary function.^1^ Predicted pulmonary function is based on values derived from healthy individuals, defined as those without a history of tobacco use or respiratory symptoms. European Respiratory Society (ERS) and American Thoracic Society (ATS) guidelines^2^ recommend the calculation of predicted pulmonary function on the basis of age, standing height, sex, and race using reference equations developed by the Global Lung Function Initiative (GLI) in 2012.^3^ In the data used to develop these reference equations, Black individuals were found to have lower lung function than their White counterparts when controlling for age, sex, and standing height.^3,4^ The use of these reference equations to predict normal lung function thus leads to the conclusion that a lower level of lung function among Black individuals is normal.

The continued use of race in clinical prediction models has been the subject of substantial recent debate.^5,6,7,8^ As race is a sociocultural construct without biological basis,^9,10,11^ observed racial differences in health reflect systemic racism and discrimination rather than innate genetic differences between racial or ethnic groups. The inclusion of race in prediction models may therefore function to mask the consequences of systemic racism, perpetuate biological essentialism, and ultimately widen health disparities.

While several studies have questioned the use of race in PFT interpretation,^12,13,14,15,16,17,18,19,20,21^ the impact of race-specific reference equations on PFT interpretation in clinical practice remains unknown. The debate has been limited by the absence of globally representative and race-neutral reference equations for normal lung function, developed in accordance with the statistical methods used to develop the currently recommended race-specific reference equations. The new GLI Global reference equations^22^—race-neutral reference equations in the sense that they do not use race as a predictor of normal lung function—provide an opportunity to interpret PFTs without the use of patient race. However, their impact on PFT interpretation has not yet been described. Therefore, we sought to compare interpretations produced using the GLI Global reference equations with those produced using the currently recommended race-specific 2012 GLI reference equations.

## Methods

We followed the relevant portions of the Strengthening the Reporting of Observational Studies in Epidemiology (STROBE) reporting guidelines for cross-sectional studies. This study was determined to be exempt by the institutional review board of the University of Pennsylvania. Additional consent was waived, as the only link between the participant and the study would have been the consent document, and the primary risk was a breach of confidentiality.

### Study Population

We collected spirometry and lung volume measurements from all individuals for whom testing was performed at a single academic medical center between January 2010 and December 2020. We considered only those individuals for whom the 2012 GLI reference equations could be applied in a race-specific manner and for whom the necessary self-reported racial identifiers were available.^3^ As a result, only those individuals identified as Black or White were included in this study. Data were included only if both spirometry and lung volumes were measured. We included only the first available PFT performed for each individual.

### Reference Equations

We compared PFT interpretations from the race-specific 2012 GLI reference equations^3^ with the new, race-neutral GLI Global reference equations.^22^ The 2012 GLI reference equations were developed by applying the generalized additive model for location, scale, and shape (GAMLSS) framework^23^ to predict forced expiratory volume in first second of expiration (FEV_1_), forced vital capacity (FVC), and percent FVC exhaled in first second (FEV_1_/FVC) on the basis of age, sex, standing height, and race. These reference equations were developed using spirometry data from 74 187 individuals from 33 countries, all of whom reported no history of either tobacco use or respiratory symptoms. The new GLI Global reference equations were developed by applying the same statistical methods used to develop the 2012 GLI reference equations, using the same set of individuals from 33 countries, but with only age, sex, and standing height as predictors. The GLI Global reference equations thus differ from the 2012 GLI reference equations in that they do not consider race in predicting normal lung function. In developing these equations, an inverse probability weight was applied to each observation, based on the race or ethnicity of the individual within the original data. These weights functioned to increase the relative contribution of groups that were underrepresented within these data.

In a secondary analysis, PFTs were interpreted using the 2012 GLI reference equations with all individuals classified as “other.”^3^ The GLI “other” reference equations were developed from the race-specific 2012 GLI reference equations by averaging the mean and coefficient of variation values across the 4 races included in the latter. These reference equations have been used in PFT interpretation to mitigate potential racial inequities associated with the race-specific 2012 GLI reference equations.^15,18,20,24^

### Interpretive Strategies

All tests were performed in accordance with ATS/ERS standards.^25,26^ Recently updated ERS/ATS guidelines were used for PFT interpretation.^2^ For each individual, the lower limits of normal (fifth centiles) of the FEV_1_, FVC, and FEV_1_/FVC were calculated using both the race-specific 2012 GLI reference equations and the race-neutral GLI Global reference equations. The lower limit of normal for the total lung capacity (TLC) was calculated using the 2021 GLI reference equations for TLC.^27^ These reference equations were developed using lung volume data from White individuals only. For a given set of reference equations, a test was interpreted as obstructive if the measured FEV_1_/FVC was less than the lower limit of normal, as restrictive if both the measured FVC and the measured TLC were less than the lower limits of normal, as mixed if both obstructive and restrictive criteria were met, as nonspecific if both FEV_1_/FVC and TLC were greater than the lower limit of normal and FVC or FEV_1_ was less than the lower limit of normal, and as normal otherwise.

Severity was assessed in accordance with ERS/ATS guidelines using FEV_1_ *z* scores.^2^ A *z* score greater than −1.645 was considered normal, while a *z* score between −1.645 and −2.5 was considered mild, a *z* score between −2.5 and −4.0 was considered moderate, and a *z* score less than −4.0 was considered severe.

### Outcomes

Primary outcomes were the changes in the percentages of individuals with obstructive, restrictive, mixed, and nonspecific pulmonary impairments when PFTs were interpreted using the race-specific 2012 GLI reference equations, compared with the race-neutral GLI Global reference equations. Secondary outcomes were the changes in severity when PFTs were interpreted with these 2 sets of reference equations. In a secondary analysis we further compared these interpretations with those produced using the 2012 GLI reference equations to calculate *z* scores, with the race of each individual classified as other.

### Statistical Analysis

Multivariable logistic regression was used to estimate the association between age, sex, height, and race and changes in the interpretations produced using the GLI Global reference equations and 2012 GLI reference equations, representing either the presence of a new respiratory impairment or an increase in severity. All analyses were performed using the R version 4.2.1 (R Project for Statistical Computing).

## Results

**Figure 1.**
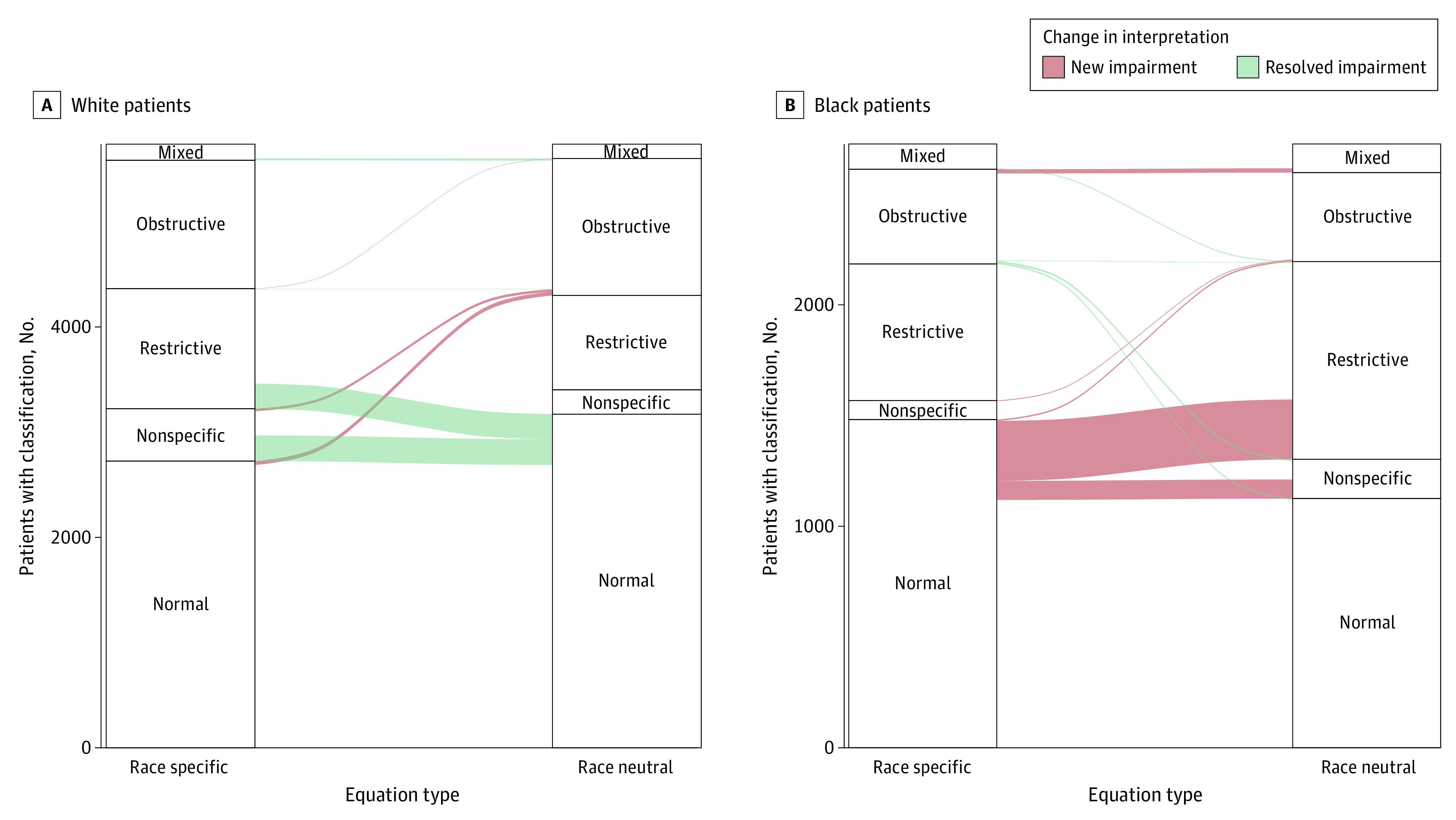
Association of Race-Neutral Reference Equations With the Identification of Obstructive, Restrictive, Mixed, and Nonspecific Respiratory Impairments Alluvial plots depict changes in the interpretation of White (A) and Black (B) individuals as having mixed, obstructive, restrictive, and nonspecific respiratory impairments and normal lung function when comparing the race-specific 2012 Global Lung Function Initiative reference equations and the race-neutral Global Lung Function Initiative Global reference equations. Strata within each axis represent the interpretations resulting from the application of American Thoracic Society/European Respiratory Society guidelines with the race-specific reference equations and the race-neutral Global reference equations, respectively. Alluvia between axes represent changes in interpretation between the 2 sets of reference equations. Red alluvia represent individuals for whom the use of the race-neutral reference equations resulted in a new respiratory impairment while green flows represent the resolution of a respiratory impairment.

PFTs from 8431 individuals were interpreted, with 2722 Black (686 men [25.4%]; mean [SD] age 51.8 [13.9] years) and 5709 White (2654 men [46.5%]; mean [SD] age 56.4 [14.3] years) individuals (Table 1). Among Black individuals, replacing the race-specific 2012 GLI reference equations with the race-neutral GLI Global reference equations was associated with 291 new cases (10.7%; 95% CI, 9.5%-11.9%) of restriction, 9 new cases (0.3%; 95% CI, 0.1%-0.5%) of obstruction, and 94 new cases (3.5%; 95% CI, 2.8%-4.1%) of nonspecific impairment. At the same time, obstruction was no longer seen in 20 Black individuals (0.7%; 95% CI, 0.4%-1.1%), while nonspecific impairments were no longer seen in 3 Black individuals (0.1%; 95% CI, 0.0%-0.2%). Among Black individuals, no cases of restriction were reinterpreted as normal (Figure 1; eFigures 1-4 in Supplement 1). Overall, replacing the race-specific reference equations with the race-neutral reference equations was associated with an increase in the prevalence of restriction from 26.8% (95% CI, 25.2%-28.5%) to 37.5% (95% CI, 35.7%-39.3%) and of a nonspecific pattern of impairment from 3.2% (95% CI, 2.5%- 3.8%) to 6.5% (95% CI, 5.6%-7.4%) and no significant change in the prevalence of obstruction (19.9% [95% CI, 18.4%-21.4%] to 19.5% [95% CI, 18.0%-21.0%]).

**Table 1.** Characteristics of Participants

Characteristic	Participants, No. (%)	
	White (n = 5709)	Black (n = 2702)
Demographic characteristics		
Age, y		
10-19	29 (1)	17 (1)
20-29	321 (6)	172 (6)
30-39	448 (8)	361 (13)
40-49	758 (13)	569 (21)
50-59	1312 (23)	761 (28)
60-69	1804 (32)	557 (21)
70-79	989 (17)	272 (10)
80-89	48 (1)	13 (1)
Sex		
Men	2654 (46)	686 (25)
Women	3055 (54)	2036 (75)
Spirometry, mean (SD)		
FEV_1_, L	2.32 (0.91)	2.01 (0.72)
FVC, L	3.19 (1.07)	2.65 (0.84)
FEV_1_/FVC, %	0.72 (0.14)	0.76 (0.13)
Lung volumes, mean (SD)		
TLC, L	5.35 (1.40)	4.54 (1.11)
ERS/ATS classification^a^		
Normal	2711 (47)	1479 (54)
Nonspecific	495 (9)	86 (3)
Obstructive	1215 (21)	427 (16)
Restrictive	1136 (20)	616 (23)
Mixed	152 (3)	114 (4)
ERS/ATS severity^a^		
Normal	3082 (54)	1630 (60)
Mild	1091 (19)	549 (20)
Moderate	1116 (20)	462 (17)
Severe	420 (7)	81 (3)

Abbreviations: ATS, American Thoracic Society; ERS, European Respiratory Society; FEV_1_, forced expiratory volume in first second of expiration; FVC, forced vital capacity; GLI, Global Lung Function Initiative; TLC, total lung capacity.

^a^ Applying the race-specific 2012 GLI reference equations with current ERS/ATS guidelines.

Among White individuals, the use of the GLI Global reference equations was associated with 64 new cases (1.1%; 95% CI, 0.8%-1.4%) of obstruction and no new cases of restriction or the nonspecific pattern. Restriction was no longer seen in 259 White individuals (4.5%; 95% CI, 4.0%-5.1%), and the nonspecific pattern was no longer seen in 265 White individuals (4.6%; 95% CI, 4.1%-5.2%). Among White individuals, no cases of obstruction were reinterpreted as normal. Overall, replacing the race-specific reference equations with the race-neutral reference equations was associated with a decrease in the prevalence of restriction from 22.6% (95% CI, 21.5%-23.6%) to 18.0% (95% CI, 17.0%-19.0%), a decrease in the prevalence of a nonspecific pattern of impairment from 8.7% (95% CI, 7.9%-9.4%) to 4.0% (95% CI, 3.5%-4.5%), and no significant change in the percentage with obstruction from 23.9% (95% CI, 22.8%-25.1%) to 25.1% (95% CI, 23.9%- 26.2%).

**Figure 2.**
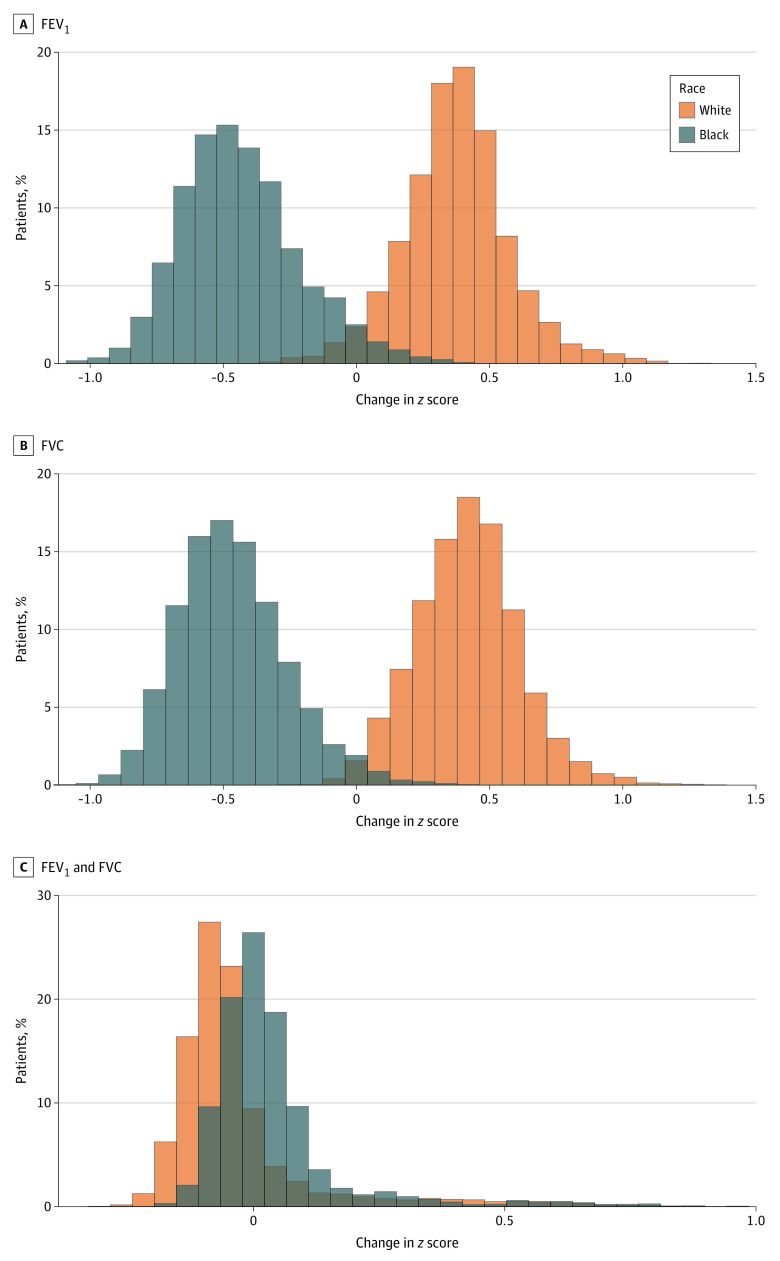
Association of Race-Neutral Reference Equations With Forced Expiratory Volume in First Second of Expiration (FEV_1_), Forced Vital Capacity (FVC), and Percent FVC Exhaled in the First Second (FEV_1_/FVC) *z* Scores in Black and White Individuals Differences in the *z* scores of FEV_1_ (A), FVC (B), and FEV_1_/FVC (C) among Black and White individuals when applying the race-specific 2012 Global Lung Function Initiative reference equations and the race-neutral Global Lung Function Initiative Global reference equations. An increase in *z* score represents an increase in apparent pulmonary function, while a decrease represents a decline in apparent pulmonary function.

Among Black individuals, replacing the race-specific 2012 GLI reference equations with the race-neutral GLI Global reference equations was associated with a mean decrease in the FEV1 *z* score of 0.43 (95% CI, 0.43-0.44) and a mean decrease in the FVC *z* score of 0.46 (95% CI, 0.46-0.47). Among White individuals, the GLI Global reference equations was associated with a mean increase in the FEV1 *z* score of 0.38 (95% CI, 0.37-0.38) and a mean increase in FVC *z* score of 0.42 (95% CI, 0.41-0.42). The adoption of the GLI Global reference equations were associated with less change in the FEV_1_/FVC *z* score, with a mean increase of 0.04 (95% CI, 0.03-0.04) for Black individuals and a mean decrease of 0.03 (95% CI, 0.03-0.04) for White individuals (Figure 2).

**Figure 3.**
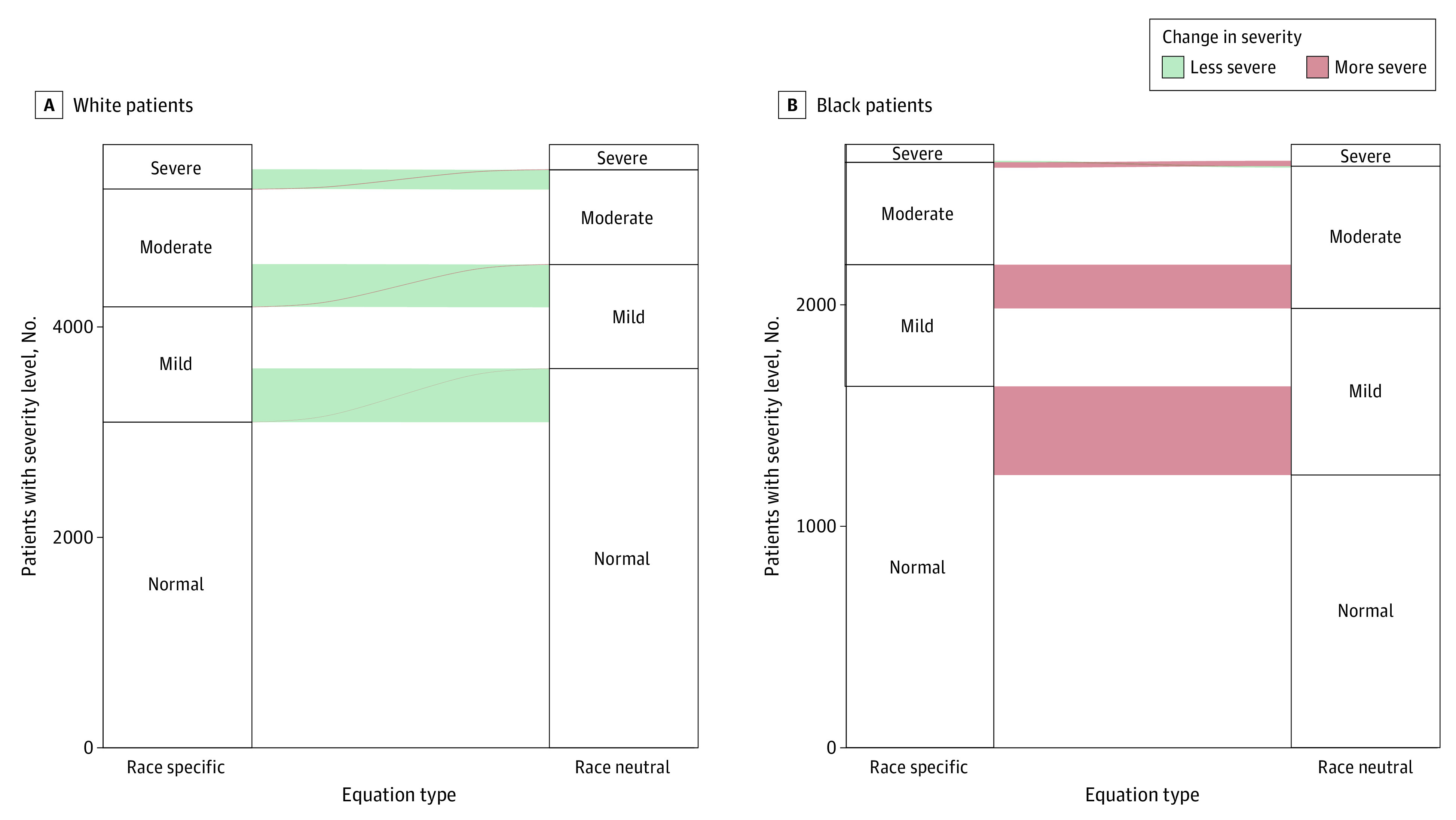
Association of Race-Neutral Reference Equations With the Severity of Lung Function Impairment Alluvial plots depict changes in the severity of lung function impairment in White (A) and Black (B) individuals. Strata within each axis represent the number of individuals with severe, moderate, mild, and normal lung function impairments when applying the race-specific 2012 Global Lung Function Initiative reference equations and the race-neutral Global Lung Function Initiative Global reference equations, respectively. Alluvia between axes represent differences in severity between the 2 sets of reference equations. Red alluvia represent a more severe impairment, while green alluvia represent a less severe impairment.

Among Black individuals, the GLI Global reference equations were associated with an increase in the severity of disease in 621 individuals (22.8%; 95% CI, 21.2%-24.4%) and a decrease in severity in 7 individuals (0.3%; 95% CI, 0.1%-0.4%). Among White individuals, the GLI Global reference equations was associated with a decrease in the severity of disease in 1100 individuals (19.3%; 95% CI, 18.2%-20.3%) and an increase in severity in 10 individuals (0.2%; 95% CI, 0.1%-0.3%). (Figure 3; eFigures 5-8 in Supplement 1).

Replacing the race-specific 2012 GLI reference equations with the GLI Global reference equations was associated with changes in PFT interpretation—representing either a change in the classification of an individual’s pulmonary function as normal, obstructive, restrictive, or nonspecific, or a change in disease severity—in 820 Black individuals (30.1%; 95% CI, 28.4%-31.8%) and 1355 White individuals (23.7%; 95% CI, 22.6%-24.8%).

Height and race were significantly associated with increased odds that the GLI Global reference equation was associated with the identification of a new respiratory impairment. Black individuals were more likely than White individuals (odds ratio [OR], 15.2; 95% CI, 11.6-20.3) to have a new impairment. Age, height, sex, and race were all found to have a significant association with the odds that the GLI Global reference equations resulted in an increase in the severity of disease. Women were less likely than men (OR, 0.6; 95% CI, 0.5-0.8) and Black individuals were more likely than White individuals (OR, 237.2, 95% CI, 133.3-478.0) to have an increase in disease severity (Table 2).

**Table 2.** Association of Reference Equation Parameters With Changes in Interpretation

Characteristic	New impairment		Increased severity	
	OR (95% CI)	*P* value	OR (95% CI)	*P* value
Age, y	1.00 (0.99-1.01)	.70	1.02 (1.01-1.03)	<.001
Height, cm	1.03 (1.01-1.04)	.003	1.02 (1.01-1.04)	<.001
Sex				
Male	1 [Reference]	NA	1 [Reference]	NA
Female	1.4 (1.0-1.9)	.05	0.6 (0.5-0.8)	<.001
Race				
White	1 [Reference]	NA	1 [Reference]	NA
Black	15.2 (11.6-20.3)	<.001	237.2 (133.3-478.0)	<.001

Abbreviations: NA, not applicable; OR, odds ratio.

When compared with the race-specific 2012 GLI reference equations, the race-neutral GLI “other” reference equations were also associated with a significant increase in the number of Black individuals with restrictive and nonspecific impairments and in the severity of these impairments (eFigures 9-11 in Supplement 1). When compared with the GLI “other” reference equations, the GLI Global reference equations were found to produce fewer and less severe changes in interpretation (eFigures 12-14 in Supplement 1).

## Discussion

We applied the race-neutral GLI Global reference equations to a cohort of Black and White individuals and compared the resultant interpretations with those produced using the race-specific 2012 GLI reference equations. Applying the race-neutral reference equations led to a significant increase both in the number of Black individuals with restrictive and nonspecific respiratory impairments and in the severity of these impairments. The GLI Global reference equations had less of an association with the identification of obstruction. These findings indicate that the use of race-specific reference equations for PFT interpretation may promote health disparities by inflating the apparent lung function of Black individuals, thus obscuring impairments that would otherwise have been identified with a race-neutral approach.

The widespread adoption of the GLI Global reference equations would dramatically alter the national epidemiology of restrictive respiratory impairments among Black individuals. The large changes in *z* scores resulted in a new interpretation of restriction in more than 10% of Black individuals, and an increase in severity in more than 20% of Black individuals. The race-neutral reference equations were associated with an increase in the total number of Black individuals with restriction by nearly 40%. To contextualize these percentages, consider that pulmonary function testing is recommended for all individuals with respiratory symptoms,^28^ including the nearly 9 million Black individuals in the United States who report dyspnea on exertion, chronic cough, or wheezing according to projections from the National Health and Nutrition Examination Survey. Thus, if the population of Black individuals who underwent pulmonary function testing in this study were similar to the population of Black individuals for whom PFTs would be recommended, the use of the GLI Global reference equations at a national level would lead to almost 1 million additional cases of restrictive ventilatory impairments among Black individuals.

The use of the GLI Global reference equations would likely have significant consequences for clinical practice. While this analysis estimated the interpretive outcomes associated with the use of the GLI Global reference equations, PFT interpretation is the basis for many clinical decisions related to the testing, diagnosis, and treatment of individuals with respiratory disease.^29,30,31,32,33,34,35,36^ For example, percent predicted FEV_1_ and FVC values—precursors to the FEV_1_ and FVC *z* scores currently recommended by ERS/ATS—inform the assessment of diseases ranging from asthma^37^ and chronic obstructive pulmonary disease^38,39^ to cystic fibrosis.^40^ And specific FEV_1_ thresholds guide clinical decisions concerning medical therapy,^41^ transplant referral,^42^ and endobronchial valve placement.^43,44^ It is likely that by increasing the apparent FEV_1_ and FVC in Black individuals, while also decreasing the apparent FEV_1_ and FVC in White individuals, the use of race-specific reference equations promotes the unequal allocation of medical resources. This may also extend to screening individuals for employment opportunities. The reduction in apparent pulmonary function associated with the GLI Global reference equations may limit some of these opportunities for Black individuals, while simultaneously providing additional support for individuals whose respiratory health was adversely affected by occupational exposures.^45,46^ Further work is needed to quantify the precise impact PFT interpretation has on clinical decision-making and employment eligibility as they impact health and economic outcomes.

The interpretive consequences that follow from the continued use of race-specific reference equations, coupled with the availability of the GLI Global reference equations, offer a strong argument in support of the routine use of these race-neutral reference equations for PFT interpretation. The idea that a race-specific approach should be applied to PFT interpretation dates to the 1850s, when observed differences in the spirometry of Black and White individuals were cited in support of fundamental differences in the pulmonary physiology of different races.^47,48^ Although beliefs about essential biological differences between individuals based on a socially constructed racial category persist, a century of science has disproved them. Rather than correct for fundamental natural differences, the incorporation of racial and ethnic categories into reference equations has served to mask social, political, and economic realities under the guise of essential biology. For this reason, race-neutral reference equations for PFT interpretation, such as those recently developed for estimating kidney function,^49^ are needed for the field of pulmonology to achieve scientific rigor and equity by current standards. The continued use of race in PFT interpretation represents a form of structural racism that can begin to be addressed through the implementation of race-neutral reference equations.

### Limitations

This study has several limitations. First, this was a single-center, cross-sectional study, and the observed outcomes of race-neutral reference equations may differ in different clinical populations. However, case-mix differences are much more likely to affect the magnitudes of observed changes in interpretation, rather than their direction, and we suspect that meaningful interpretive changes would arise in nearly any context. Second, our study considered the consequences of race-neutral reference equations only among Black and White individuals and did not assess the impact these equations may have on individuals with other self-reported racial and ethnic categories. Third, while we interpreted PFTs by applying ERS/ATS guidelines in a purely algorithmic manner, in actual practice the pulmonologists reading these tests may choose to depart from these guidelines on the basis of additional clinical information not included within the FEV_1_, FVC, and FEV_1_/FVC *z* scores. Fourth, this study directly assessed only the interpretive consequences of the GLI Global reference equations and did not further assess the downstream clinical consequences that follow from such interpretation. Further work is needed to determine the impact race-specific reference equations may have in promoting changes in allocation—including both overdiagnosis and underdiagnosis—of health care resources to Black and White individuals. Fifth, although the GLI Global reference equations are race-neutral, in the sense that race is not included as a predictor, race nonetheless played an important role in the development of these reference equations. The spirometry data used to develop these equations were aggregated specifically with the purpose of producing race-specific reference equations for PFT interpretation, with data excluded if they were not labeled by race or ethnicity or if the data collected for a given race or ethnicity were deemed insufficient in number.^3^ Moreover, race was used in the development of the GLI Global reference equations to provide inverse probability weights for the individual PFT data. While the GLI Global reference equations are race-neutral they are not race-blind.

## Conclusions

In this study we used newly developed race-neutral reference equations to interpret PFTs and compared the resulting interpretations with those produced using the current recommended race-specific reference equations. We found that the race-neutral reference equations led to a significant increase in both the prevalence and severity of respiratory impairments among Black individuals. These findings indicate that the choice of race-specific or race-neutral reference equations were associated with PFT interpretation and that the use of race-specific reference equations may play an important role in promoting racial disparities in the diagnosis and evaluation of respiratory disease.

## Notes

Supplement 1. **eFigure 1.** Reclassification Tables Showing Differences in the Interpretation of Obstructive, Restrictive, Mixed and Nonspecific Impairments Applying the Race-Specific 2012 Global Lung Function Initiative (GLI) Model and the GLI 2012 Model to Black and White Individuals **eFigure 2.** Bar Graphs Showing Differences in the Interpretation of Obstructive, Restrictive, Mixed and Nonspecific Impairments Applying the Race-Specific 2012 Global Lung Function Initiative (GLI) Model and the GLI 2012 Model to Black and White Individuals **eFigure 3.** Alluvial Plots Showing Differences in the Interpretation of Obstructive, Restrictive, Mixed and Nonspecific Impairments Applying the Race-Specific 2012 Global Lung Function Initiative (GLI) Model and the GLI 2012 Model to Black and White Men **eFigure 4.** Alluvial Plots Showing Differences in the Interpretation of Obstructive, Restrictive, Mixed and Nonspecific Impairments Applying the Race-Specific 2012 Global Lung Function Initiative (GLI) Model and the GLI 2012 Model to Black and White Women **eFigure 5.** Reclassification Tables Showing Differences in the Severity Associated With Application of the Race-Specific 2012 Global Lung Function Initiative (GLI) Model and the Race-Neutral GLI Global Model to Black and White Individuals **eFigure 6.** Bar Graphs Showing Differences in the Severity Associated With Application of the Race-Specific 2012 Global Lung Function Initiative (GLI) Model and the Race-Neutral GLI Global Model to Black and White Individuals **eFigure 7.** Alluvial Plots Showing Differences in the Severity Associated With Application of the Race-Specific 2012 Global Lung Function Initiative (GLI) Model and the Race-Neutral GLI Global Model to Black and White Men **eFigure 8.** Alluvial Plots Showing Differences in the Severity Associated With Application of the Race-Specific 2012 Global Lung Function Initiative (GLI) Model and the Race-Neutral GLI Global Model to Black and White Women **eFigure 9.** Differences in *z* Scores Between the Race-Specific 2012 Global Lung Function Initiative Model and the GLI Other Model in Black and White Individuals **eFigure 10.** Differences in the Interpretation of Obstructive, Restrictive, Mixed, and Nonspecific Impairments Applying the Race-Specific 2012 Global Lung Function Initiative (GLI) Model and the GLI Other Model to Black and White Individuals **eFigure 11.** Differences in the Severity of Pulmonary Impairments Applying the Race-Specific 2012 Global Lung Function Initiative (GLI) Model and the GLI Other Model to Black and White Individuals **eFigure 12.** Differences in the Interpretation of Obstructive, Restrictive, Mixed and Nonspecific Impairments Applying the Global Lung Function Initiative (GLI) Global Model and the GLI Other Model to Black and White Individuals **eFigure 13.** Differences in z Scores Between the Global Lung Function Initiative (GLI) Global Model and the GLI Other Model in Black and White Individuals **eFigure 14.** Differences in the Severity of Pulmonary Impairments Applying the Global Lung Function Initiative (GLI) Global Model and the GLI Other Model to Black and White Individuals

Supplement 2.
Data Sharing Statement
