# Supplementary material for: Global, Race-Neutral Reference Equations and Pulmonary Function Test Interpretation
Source: JAMA Netw Open. 2023 Jun 1;6(6):e2316174. doi: 10.1001/jamanetworkopen.2023.16174 (PMC10236239; doi:10.1001/jamanetworkopen.2023.16174)
Supplement: Supplement 1. — eFigure 1. Reclassification Tables Showing Differences in the Interpretation of Obstructive, Restrictive, Mixed and Nonspecific Impairments Applying the Race-Specific 2012 Global Lung Function Initiative (GLI) Model and the GLI 2012 Model to Black and White Individuals eFigure 2. Bar Graphs Showing Differences in the Interpretation of Obstructive, Restrictive, Mixed and Nonspecific Impairments Applying the Race-Specific 2012 Global Lung Function Initiative (GLI) Model and the GLI 2012 Model to Black and White Individuals eFigure 3. Alluvial Plots Showing Differences in the Interpretation of Obstructive, Restrictive, Mixed and Nonspecific Impairments Applying the Race-Specific 2012 Global Lung Function Initiative (GLI) Model and the GLI 2012 Model to Black and White Men eFigure 4. Alluvial Plots Showing Differences in the Interpretation of Obstructive, Restrictive, Mixed and Nonspecific Impairments Applying the Race-Specific 2012 Global Lung Function Initiative (GLI) Model and the GLI 2012 Model to Black and White Women eFigure 5. Reclassification Tables Showing Differences in the Severity Associated With Application of the Race-Specific 2012 Global Lung Function Initiative (GLI) Model and the Race-Neutral GLI Global Model to Black and White Individuals eFigure 6. Bar Graphs Showing Differences in the Severity Associated With Application of the Race-Specific 2012 Global Lung Function Initiative (GLI) Model and the Race-Neutral GLI Global Model to Black and White Individuals eFigure 7. Alluvial Plots Showing Differences in the Severity Associated With Application of the Race-Specific 2012 Global Lung Function Initiative (GLI) Model and the Race-Neutral GLI Global Model to Black and White Men eFigure 8. Alluvial Plots Showing Differences in the Severity Associated With Application of the Race-Specific 2012 Global Lung Function Initiative (GLI) Model and the Race-Neutral GLI Global Model to Black and White Women eFigure 9. Differences in z Scores Between the Race-Specif [file jamanetwopen-e2316174-s001.pdf]

Supplemental Online Content

Moffett AT, Bowerman C, Stanojevic S, Eneanya ND, Halpern SD, Weissman GE. Global, race-neutral reference equations and pulmonary function test interpretation. *JAMA Netw Open.* 2023;6(6):e2316174. doi:10.1001/jamanetworkopen.2023.16174

eFigure 1. Reclassification Tables Showing Differences in the Interpretation of Obstructive, Restrictive, Mixed and Nonspecific Impairments Applying the Race-Specific 2012 Global Lung Function Initiative (GLI) Model and the GLI 2012 Model to Black and White Individuals

eFigure 2. Bar Graphs Showing Differences in the Interpretation of Obstructive, Restrictive, Mixed and Nonspecific Impairments Applying the Race-Specific 2012 Global Lung Function Initiative (GLI) Model and the GLI 2012 Model to Black and White Individuals

eFigure 3. Alluvial Plots Showing Differences in the Interpretation of Obstructive, Restrictive, Mixed and Nonspecific Impairments Applying the Race-Specific 2012 Global Lung Function Initiative (GLI) Model and the GLI 2012 Model to Black and White Men

eFigure 4. Alluvial Plots Showing Differences in the Interpretation of Obstructive, Restrictive, Mixed and Nonspecific Impairments Applying the Race-Specific 2012 Global Lung Function Initiative (GLI) Model and the GLI 2012 Model to Black and White Women

eFigure 5. Reclassification Tables Showing Differences in the Severity Associated With Application of the Race-Specific 2012 Global Lung Function Initiative (GLI) Model and the Race-Neutral GLI Global Model to Black and White Individuals

eFigure 6. Bar Graphs Showing Differences in the Severity Associated With Application of the Race-Specific 2012 Global Lung Function Initiative (GLI) Model and the Race-Neutral GLI Global Model to Black and White Individuals

eFigure 7. Alluvial Plots Showing Differences in the Severity Associated With Application of the Race-Specific 2012 Global Lung Function Initiative (GLI) Model and the Race-Neutral GLI Global Model to Black and White Men

eFigure 8. Alluvial Plots Showing Differences in the Severity Associated With Application of the Race-Specific 2012 Global Lung Function Initiative (GLI) Model and the Race-Neutral GLI Global Model to Black and White Women

eFigure 9. Differences in *z* Scores Between the Race-Specific 2012 Global Lung Function Initiative Model and the GLI Other Model in Black and White Individuals

eFigure 10. Differences in the Interpretation of Obstructive, Restrictive, Mixed, and Nonspecific Impairments Applying the Race-Specific 2012 Global Lung Function Initiative (GLI) Model and the GLI Other Model to Black and White Individuals

eFigure 11. Differences in the Severity of Pulmonary Impairments Applying the Race-Specific 2012 Global Lung Function Initiative (GLI) Model and the GLI Other Model to Black and White Individuals

eFigure 12. Differences in the Interpretation of Obstructive, Restrictive, Mixed and Nonspecific Impairments Applying the Global Lung Function Initiative (GLI) Global Model and the GLI Other

© 2023 Moffett AT et al. *JAMA Network Open*.

Model to Black and White Individuals

eFigure 13. Differences in z Scores Between the Global Lung Function Initiative (GLI) Global Model and the GLI Other Model in Black and White Individuals

eFigure 14. Differences in the Severity of Pulmonary Impairments Applying the Global Lung Function Initiative (GLI) Global Model and the GLI Other Model to Black and White Individuals

This supplemental material has been provided by the authors to give readers additional information about their work.

© 2023 Moffett AT et al. *JAMA Network Open*.

eFigure 1. Reclassification Tables Showing Differences in the Interpretation of Obstructive, Restrictive, Mixed and Nonspecific Impairments Applying the Race-Specific 2012 Global Lung Function Initiative (GLI) Model and the GLI 2012 Model to Black and White Individuals

A

White Patients with GLI Global (columns); White Patients with GLI 2012 (rows)

	Normal	Non-Specific	Obstructive	Restrictive	Mixed
Normal	**2675** *46.9%*	**0** *0.0%*	**36** *0.6%*	**0** *0.0%*	**0** *0.0%*
Non-Specific	**242** *4.2%*	**230** *4.0%*	**23** *0.4%*	**0** *0.0%*	**0** *0.0%*
Obstructive	**0** *0.0%*	**0** *0.0%*	**1215** *21.3%*	**0** *0.0%*	**0** *0.0%*
Restrictive	**238** *4.2%*	**0** *0.0%*	**3** *0.1%*	**893** *15.6%*	**2** *0.0%*
Mixed	**0** *0.0%*	**0** *0.0%*	**18** *0.3%*	**0** *0.0%*	**134** *2.3%*

B

Black Patients with GLI Global (columns); Black Patients with GLI 2012 (rows)

	Normal	Non-Specific	Obstructive	Restrictive	Mixed
Normal	**1117** *41.0%*	**86** *3.2%*	**6** *0.2%*	**270** *9.9%*	**0** *0.0%*
Non-Specific	**0** *0.0%*	**83** *3.0%*	**3** *0.1%*	**0** *0.0%*	**0** *0.0%*
Obstructive	**6** *0.2%*	**8** *0.3%*	**392** *14.4%*	**2** *0.1%*	**19** *0.7%*
Restrictive	**0** *0.0%*	**0** *0.0%*	**0** *0.0%*	**616** *22.6%*	**0** *0.0%*
Mixed	**0** *0.0%*	**0** *0.0%*	**0** *0.0%*	**4** *0.1%*	**110** *4.0%*

Change in Classification

Resolved Impairment: 10%, 5%

0%

New Impairment: 5%, 10%

© 2023 Moffett AT et al. *JAMA Network Open*.

eFigure 2. Bar Graphs Showing Differences in the Interpretation of Obstructive, Restrictive, Mixed and Nonspecific Impairments Applying the Race-Specific 2012 Global Lung Function Initiative (GLI) Model and the GLI 2012 Model to Black and White Individuals

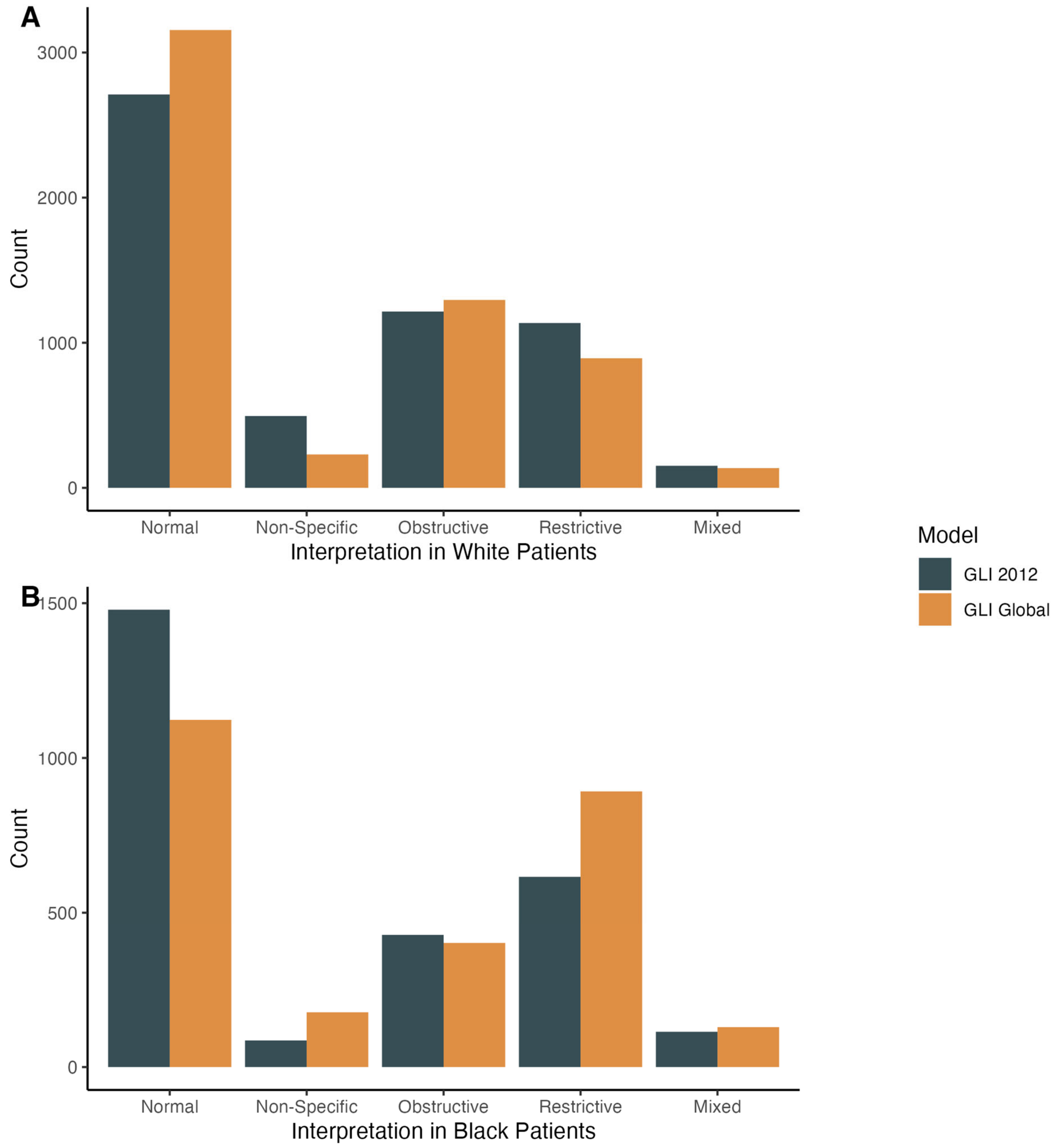

© 2023 Moffett AT et al. *JAMA Network Open*.

eFigure 3. Alluvial Plots Showing Differences in the Interpretation of Obstructive, Restrictive, Mixed and Nonspecific Impairments Applying the Race-Specific 2012 Global Lung Function Initiative (GLI) Model and the GLI 2012 Model to Black and White Men

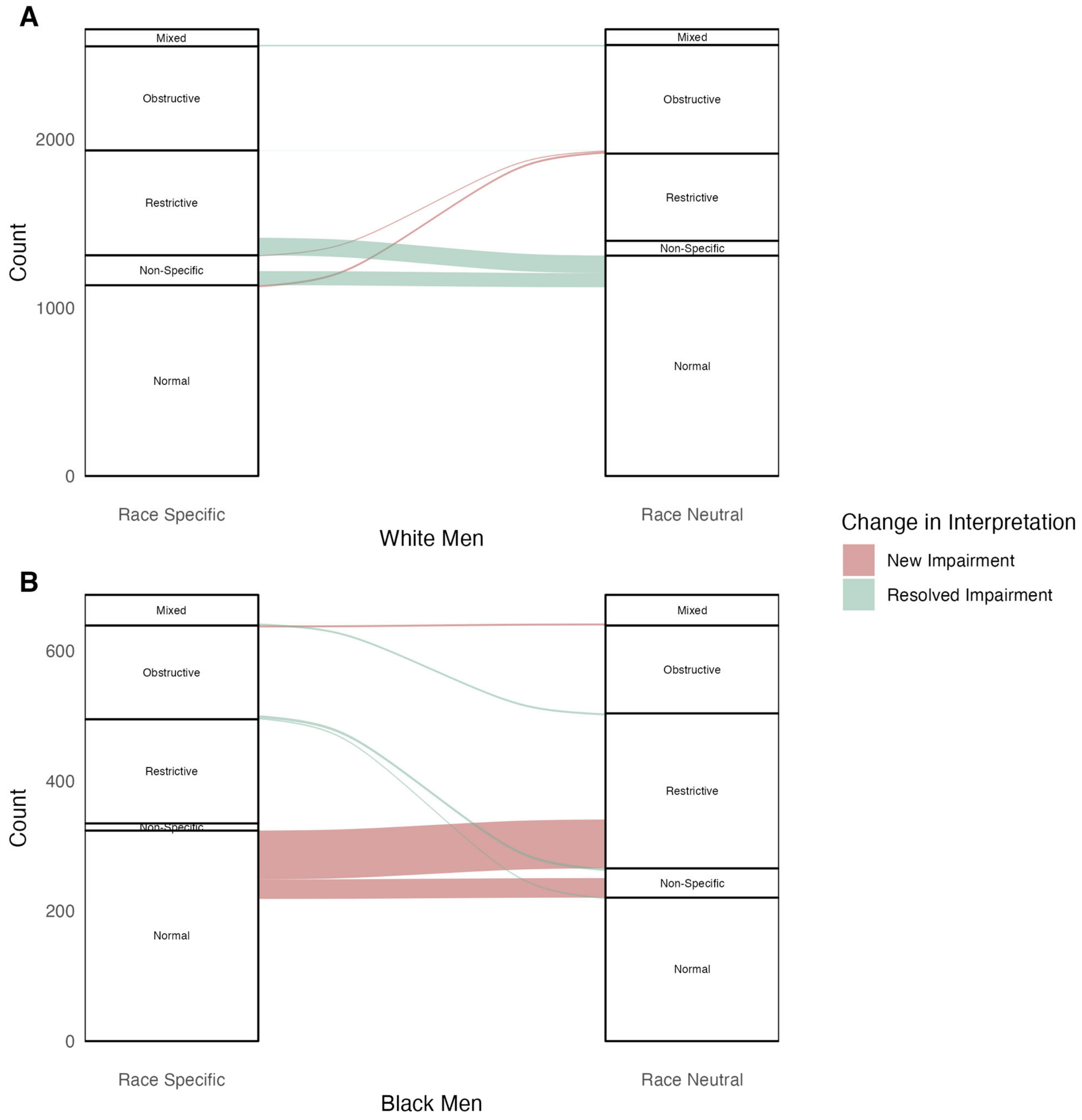

© 2023 Moffett AT et al. *JAMA Network Open*.

eFigure 4. Alluvial Plots Showing Differences in the Interpretation of Obstructive, Restrictive, Mixed and Nonspecific Impairments Applying the Race-Specific 2012 Global Lung Function Initiative (GLI) Model and the GLI 2012 Model to Black and White Women

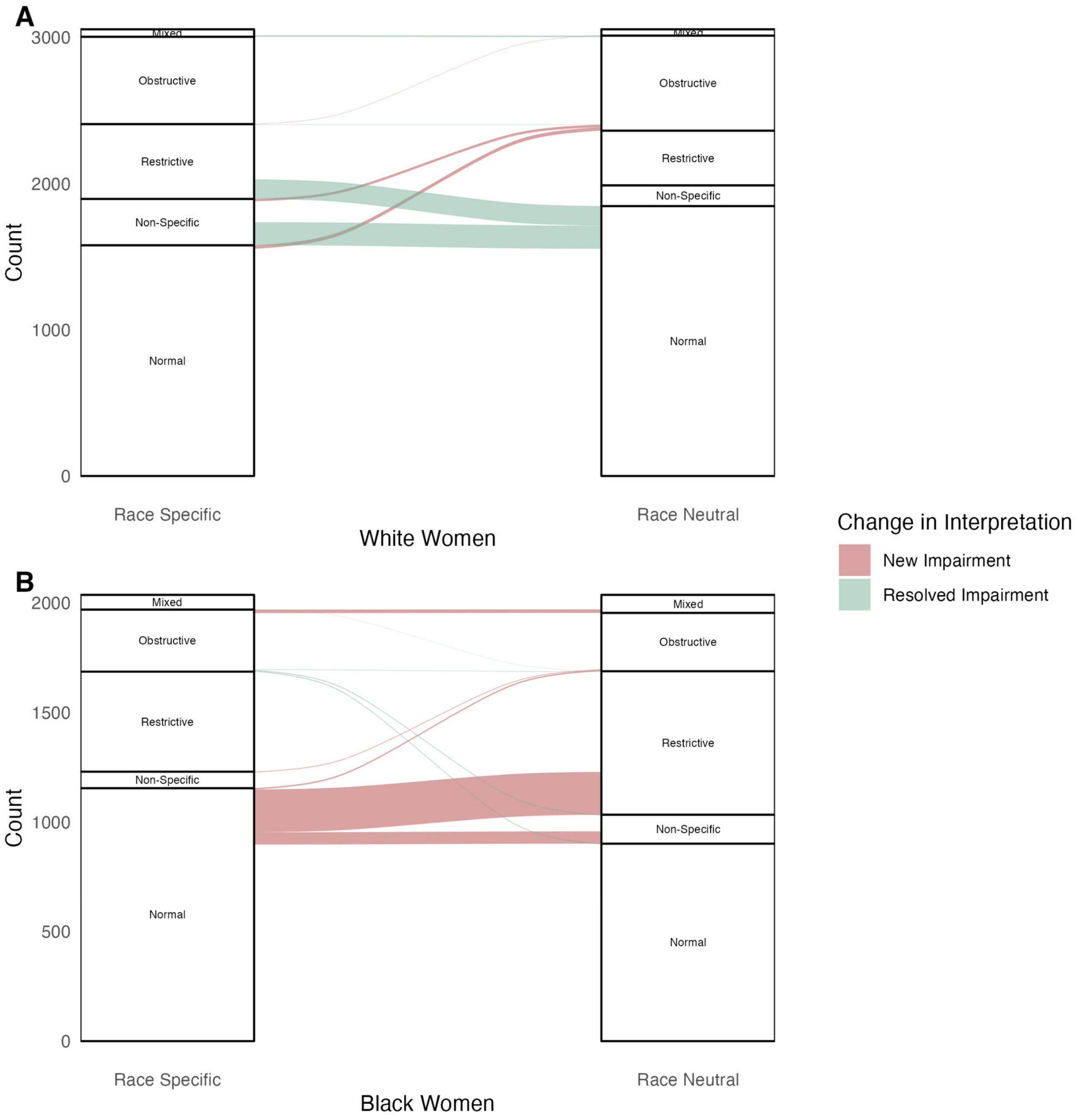

© 2023 Moffett AT et al. *JAMA Network Open*.

eFigure 5. Reclassification Tables Showing Differences in the Severity Associated With Application of the Race-Specific 2012 Global Lung Function Initiative (GLI) Model and the Race-Neutral GLI Global Model to Black and White Individuals

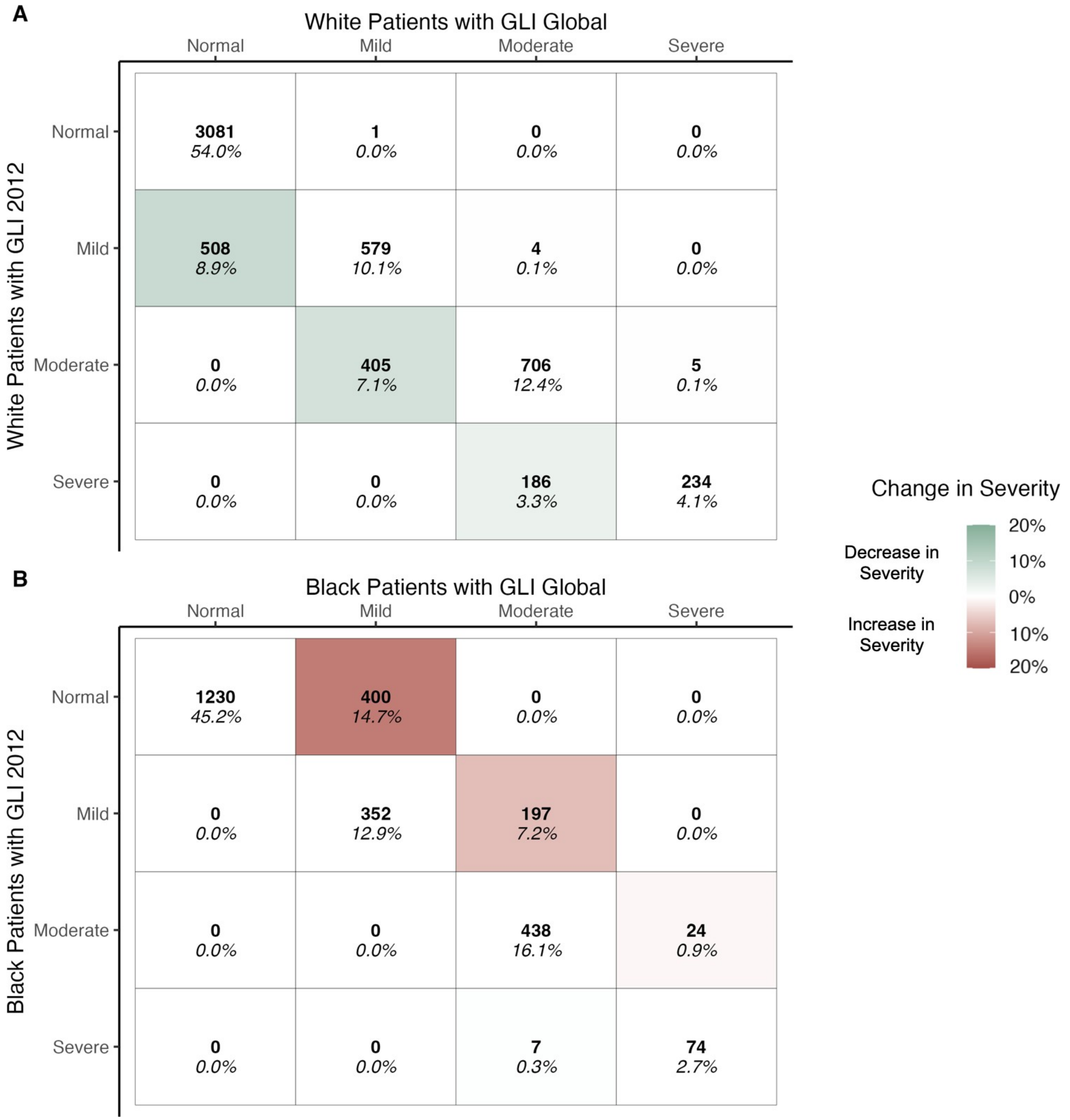

© 2023 Moffett AT et al. *JAMA Network Open*.

eFigure 6. Bar Graphs Showing Differences in the Severity Associated With Application of the Race-Specific 2012 Global Lung Function Initiative (GLI) Model and the Race-Neutral GLI Global Model to Black and White Individuals

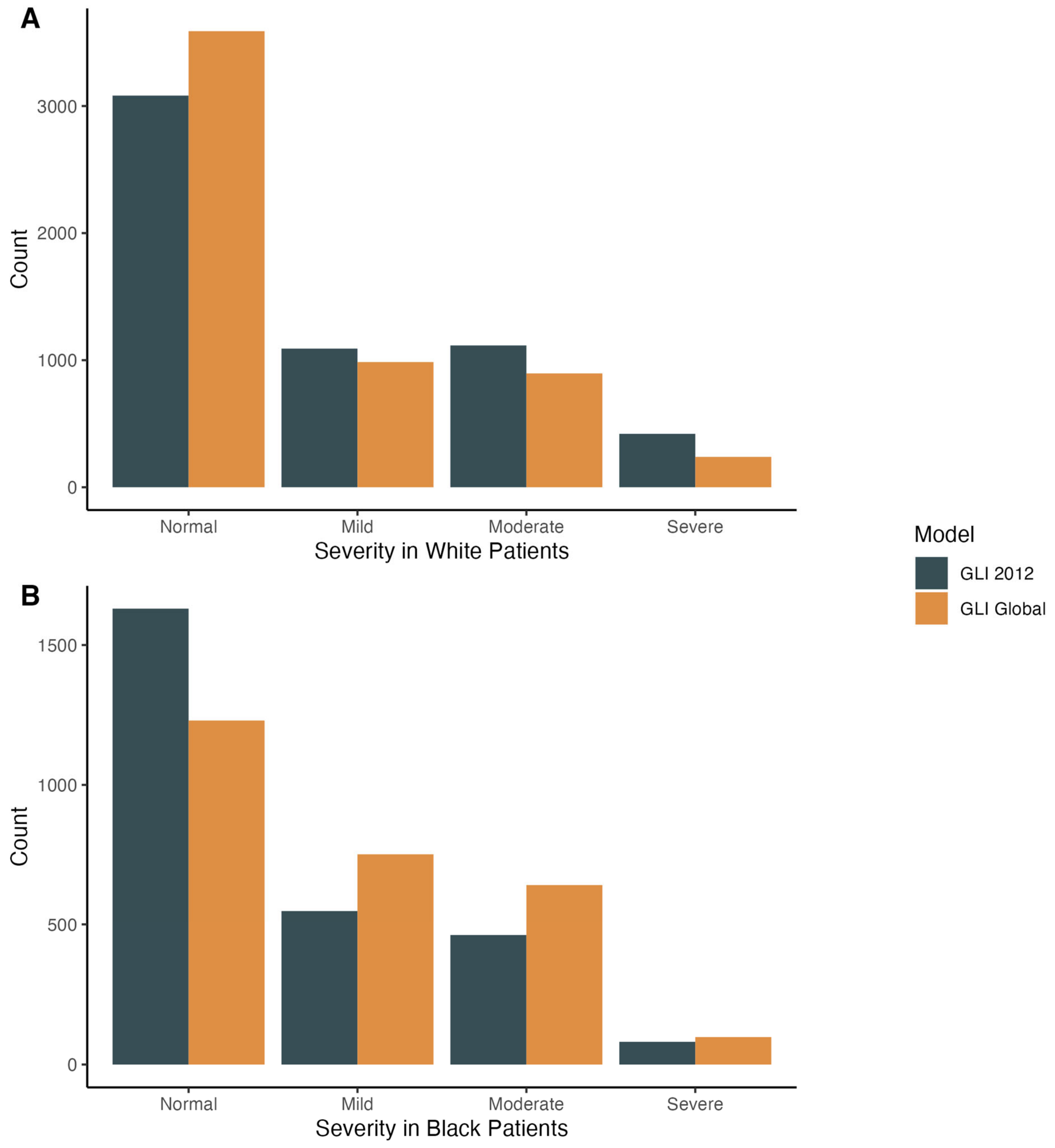

© 2023 Moffett AT et al. *JAMA Network Open*.

eFigure 7. Alluvial Plots Showing Differences in the Severity Associated With Application of the Race-Specific 2012 Global Lung Function Initiative (GLI) Model and the Race-Neutral GLI Global Model to Black and White Men

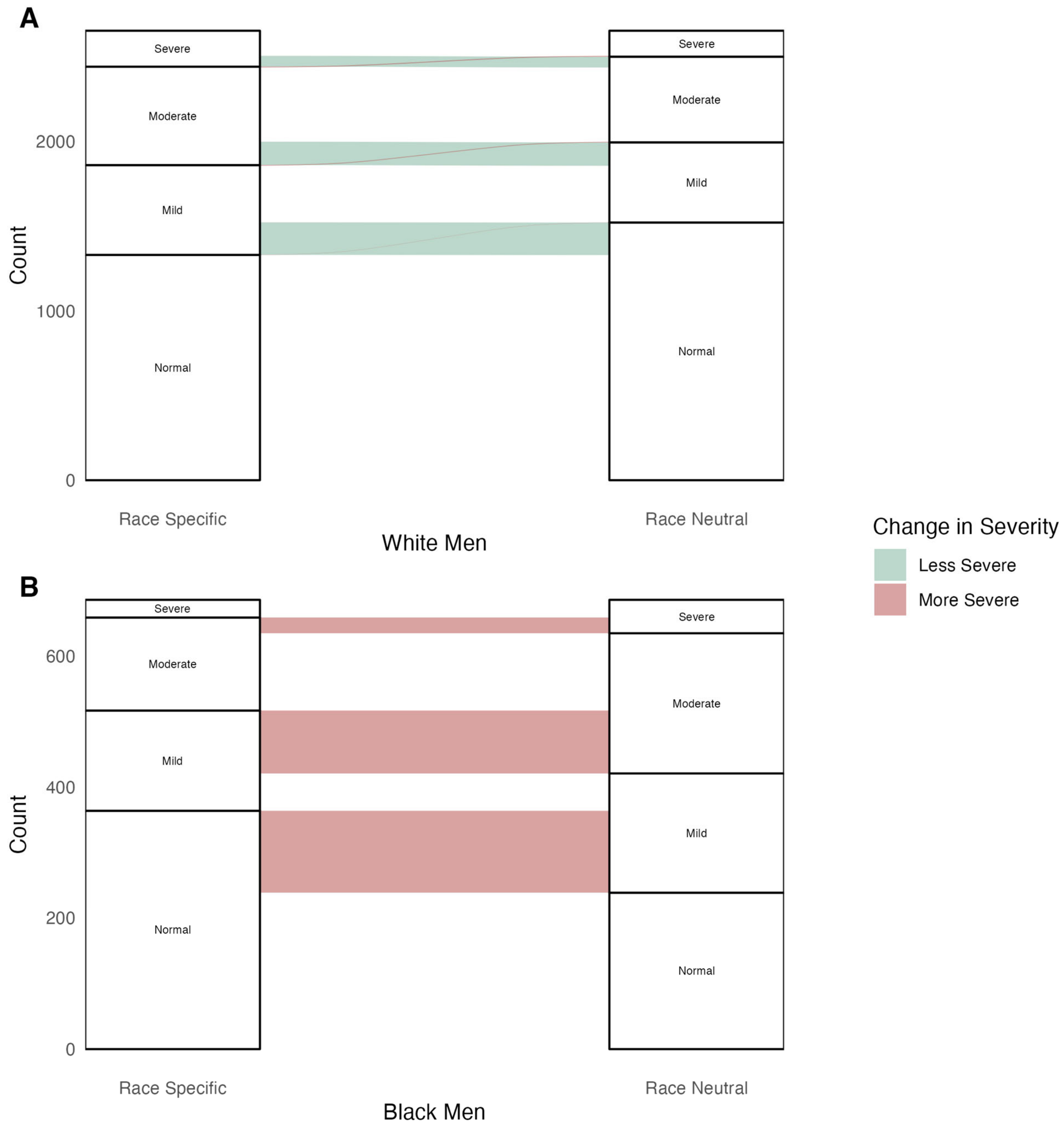

© 2023 Moffett AT et al. *JAMA Network Open*.

eFigure 8. Alluvial Plots Showing Differences in the Severity Associated With Application of the Race-Specific 2012 Global Lung Function Initiative (GLI) Model and the Race-Neutral GLI Global Model to Black and White Women

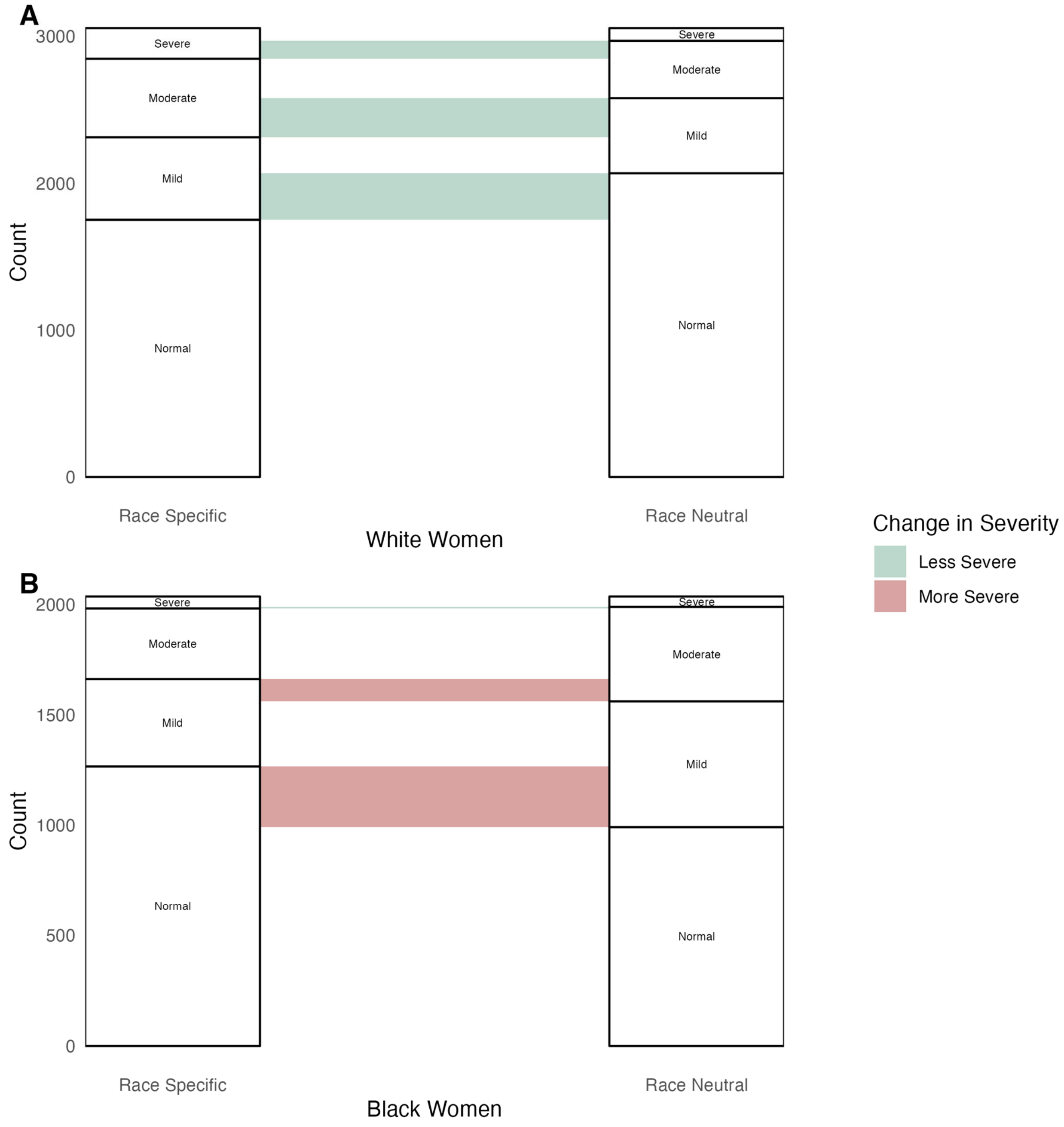

© 2023 Moffett AT et al. *JAMA Network Open*.

eFigure 9. Differences in *z* Scores Between the Race-Specific 2012 Global Lung Function Initiative Model and the GLI Other Model in Black and White Individuals

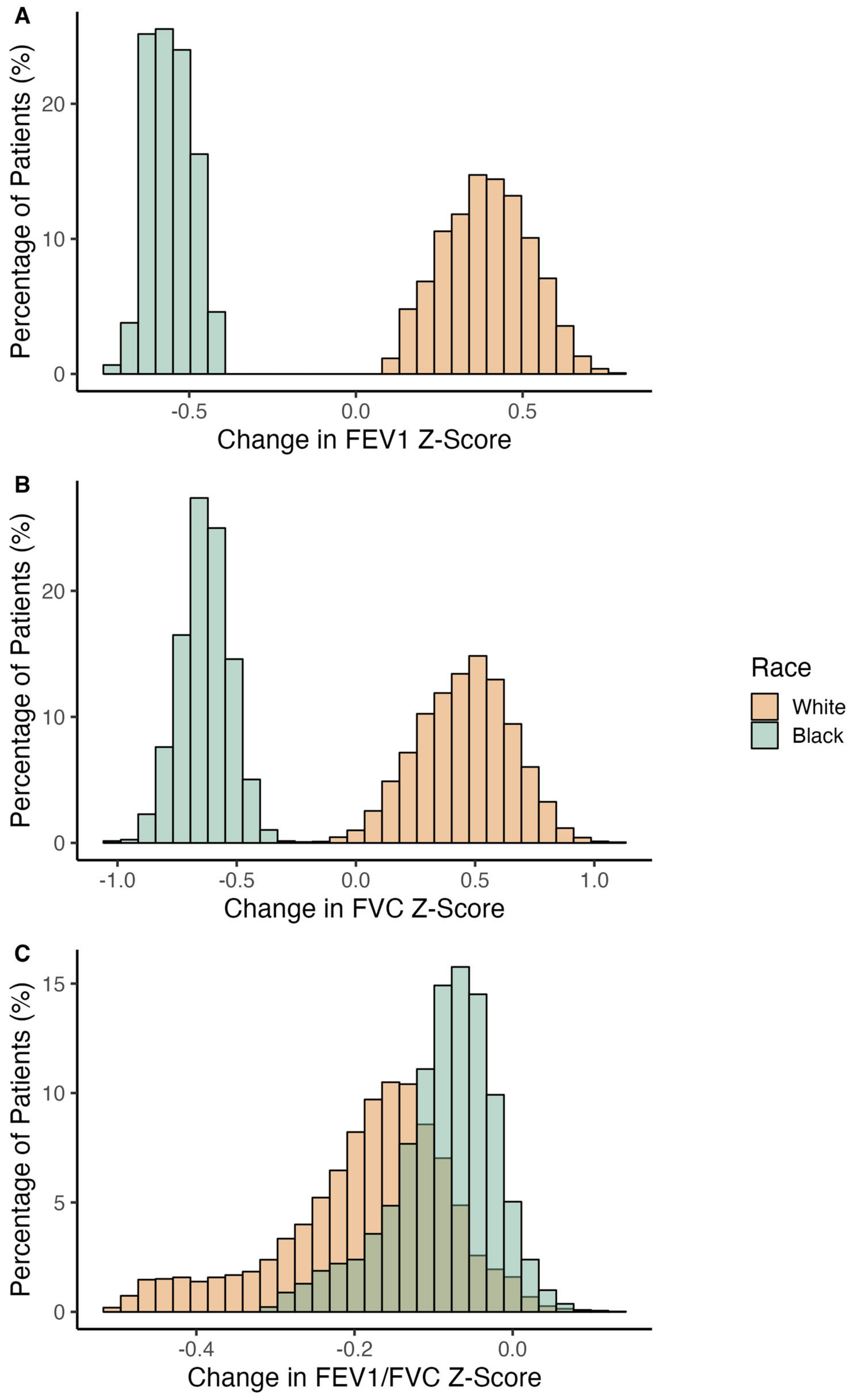

© 2023 Moffett AT et al. *JAMA Network Open*.

eFigure 10. Differences in the Interpretation of Obstructive, Restrictive, Mixed, and Nonspecific Impairments Applying the Race-Specific 2012 Global Lung Function Initiative (GLI) Model and the GLI Other Model to Black and White Individuals

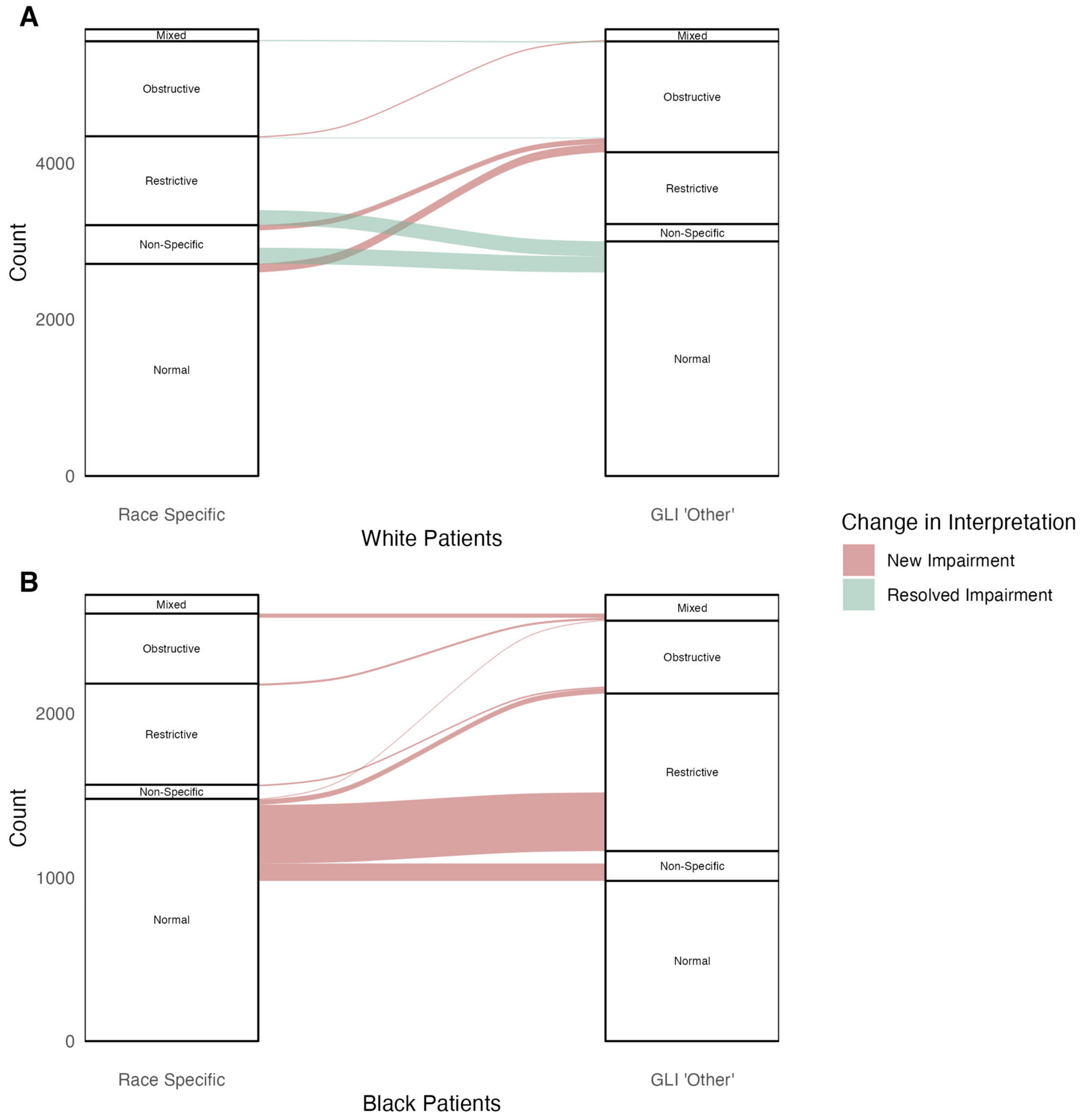

© 2023 Moffett AT et al. *JAMA Network Open*.

eFigure 11. Differences in the Severity of Pulmonary Impairments Applying the Race-Specific 2012 Global Lung Function Initiative (GLI) Model and the GLI Other Model to Black and White Individuals

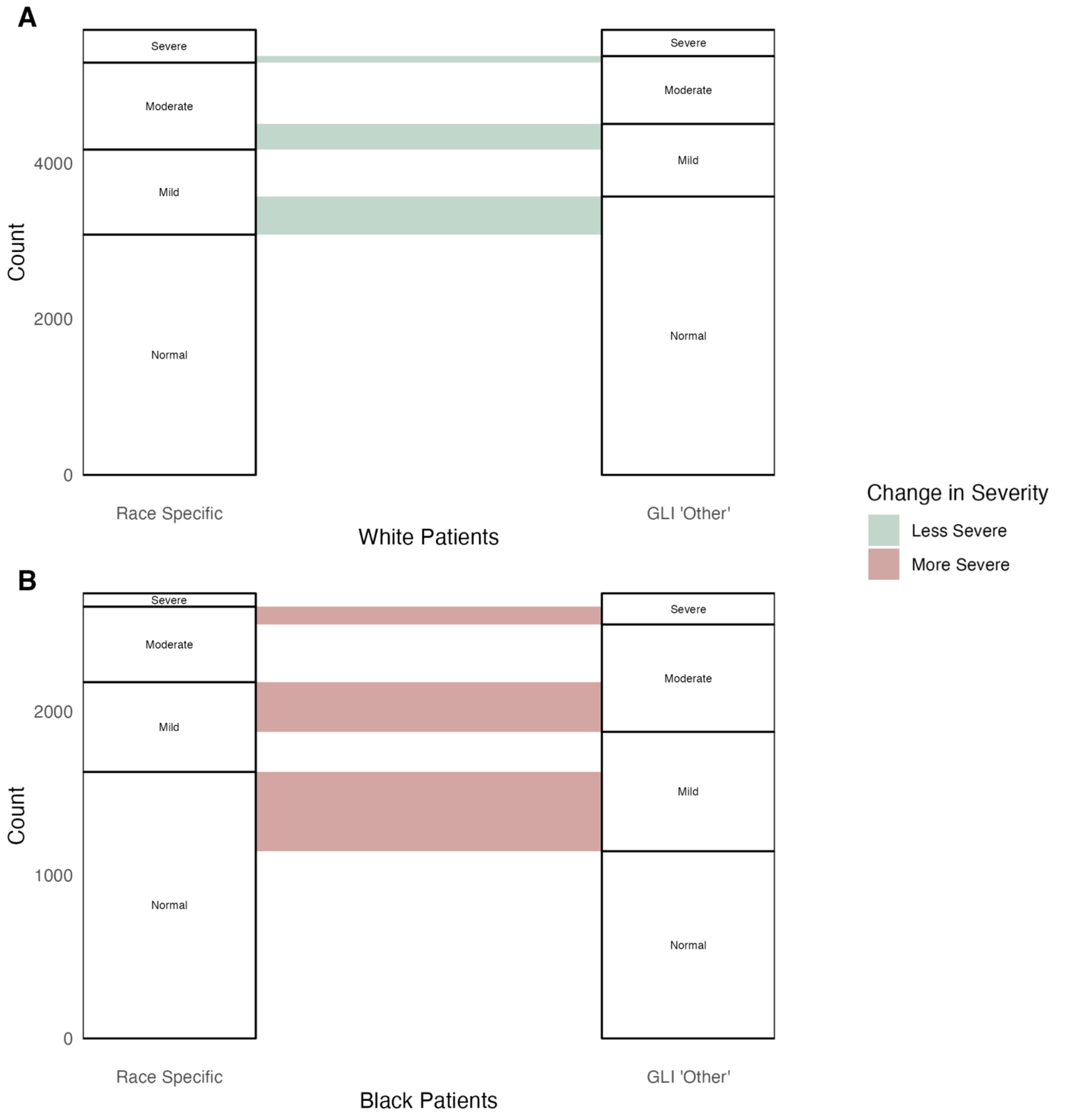

© 2023 Moffett AT et al. *JAMA Network Open*.

eFigure 12. Differences in the Interpretation of Obstructive, Restrictive, Mixed and Nonspecific Impairments Applying the Global Lung Function Initiative (GLI) Global Model and the GLI Other Model to Black and White Individuals

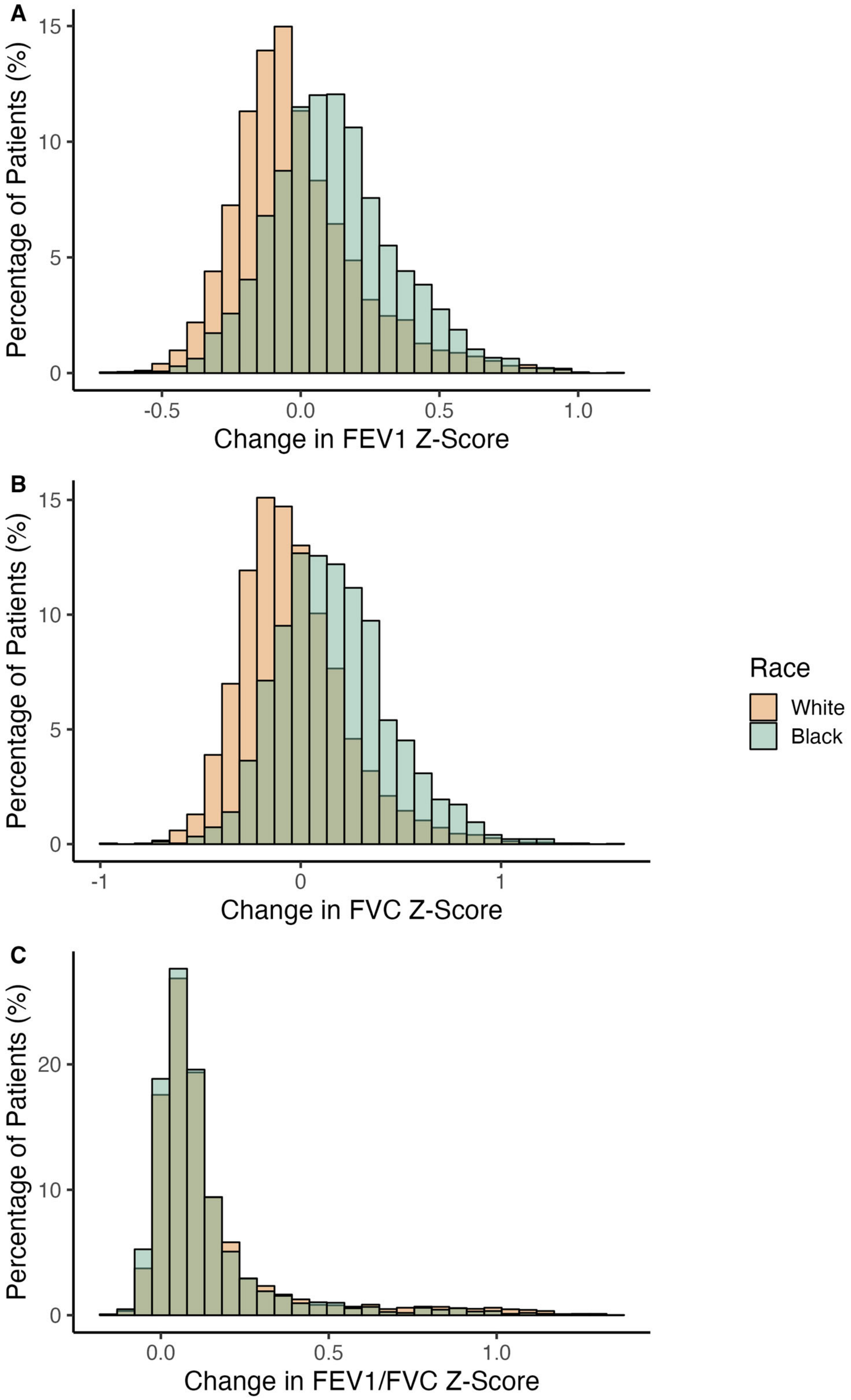

© 2023 Moffett AT et al. *JAMA Network Open*.

eFigure 13. Differences in z Scores Between the Global Lung Function Initiative (GLI) Global Model and the GLI Other Model in Black and White Individuals

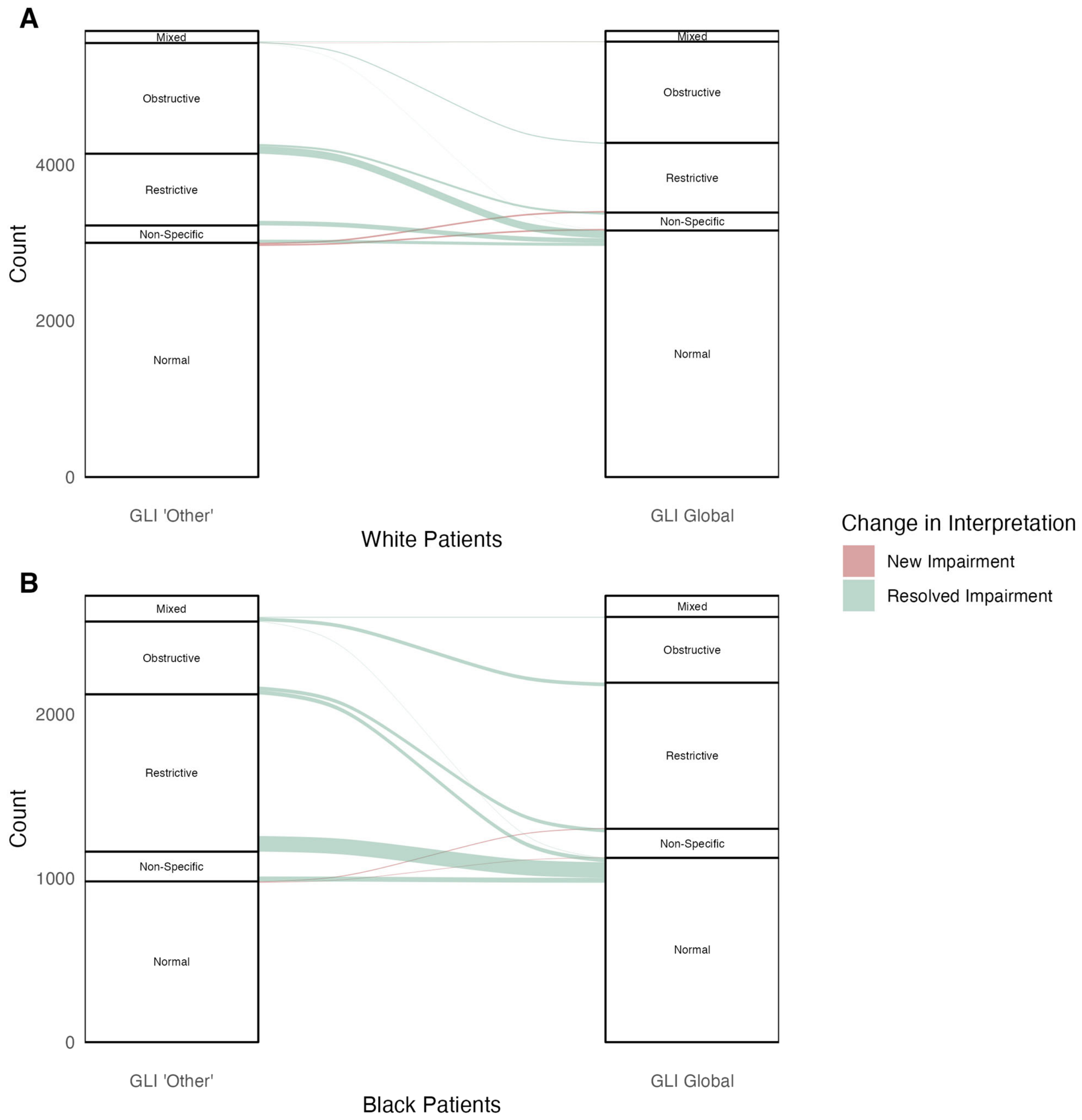

© 2023 Moffett AT et al. *JAMA Network Open*.

eFigure 14. Differences in the Severity of Pulmonary Impairments Applying the Global Lung Function Initiative (GLI) Global Model and the GLI Other Model to Black and White Individuals

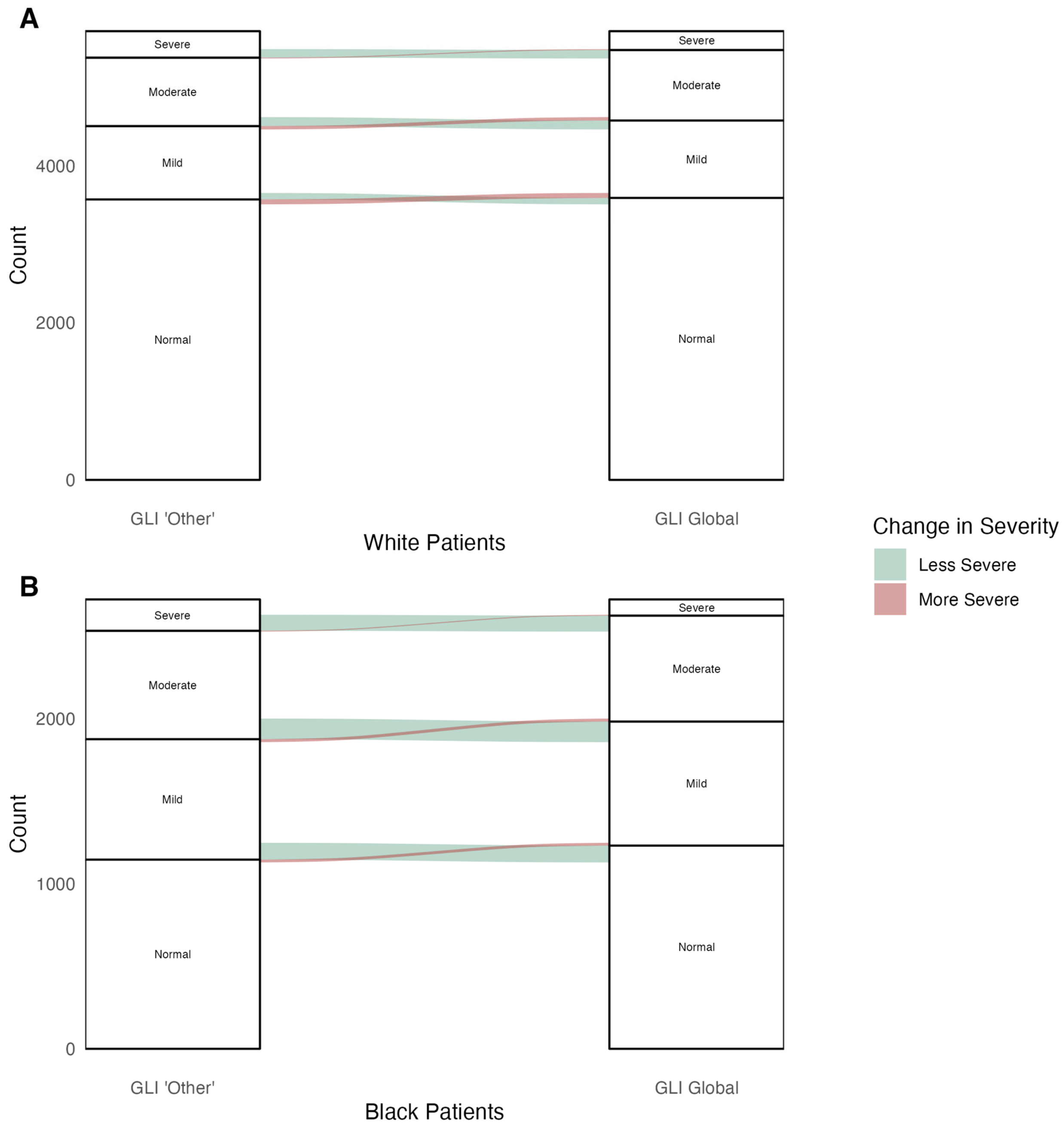

© 2023 Moffett AT et al. *JAMA Network Open*.
