# Supplementary material for: Global, Race-Neutral Reference Equations and Pulmonary Function Test Interpretation
Source: JAMA Netw Open. 2023 Jun 1;6(6):e2316174. doi: 10.1001/jamanetworkopen.2023.16174 (PMC10236239; doi:10.1001/jamanetworkopen.2023.16174)
Supplement: Supplement 2. — Data Sharing Statement [file jamanetwopen-e2316174-s002.pdf]

Data Sharing Statement

Moffett. Global, Race-Neutral Reference Equations and Pulmonary Function Test Interpretation. *JAMA Netw Open*. Published June 01, 2023. doi:10.1001/jamanetworkopen.2023.16174

Data
Data available: No
